# Opinion: Understanding EAP learners' beliefs about language learning from a socio-cultural perspective : A longitudinal study at an EMI context in mainland China

**DOI:** 10.3389/fpsyg.2022.1031767

**Published:** 2022-10-05

**Authors:** Long Qian, Zhenru Shang, Shuang Zhang

**Affiliations:** ^1^School of Humanities, Wuhan University of Engineering Science, Wuhan, China; ^2^Faculty of Humanities, Hong Kong Polytechnic University, Kowloon, Hong Kong SAR, China

**Keywords:** learner beliefs, socio-cultural perspective, ESP, EMI, language learning

The recently-published book entitled *Understanding EAP Learners' Beliefs about Language Learning from a Socio-cultural Perspective* by Li (2021) attracted our attention, for its lucid overview in synthesizing the disposition of Chinese EAP learners' beliefs about language learning (BLLs) in an EMI setting within the EFL context in Mainland China as well as the socio-cultural factors shaping and reshaping their beliefs before and after entry into the EMI environment.

This book consists of six chapters. Chapter 1 provides a comprehensive overview of the status quo and challenges of EMI learners' beliefs about English language learning. Chapter 2 first thoroughly reviews the literature on learners' beliefs, and then introduces the basics of socio-cultural theory as well as relevant concepts such as mediation and agency. After that, an analytical framework is proposed for this research. In Chapter 3, the mixed-method research design is described. In Chapter 4, the results yielded from the quantitative and qualitative data are reported. Then, the penultimate Chapter interprets the changes that took place in the participants' beliefs after they had studied EAP for an academic year at the EMI University from the socio-cultural perspective. Chapter 6 summarizes the manuscript regarding the contributions, pedagogical implications and limitations of the study as well as suggestions for future investigation. This captivating book distinguishes itself with its significant theoretical, practical, and methodological strengths.

First and foremost, this book sheds new light on beliefs about language learning from the socio-cultural approach, which breaks the domination of cognition-oriented approach in this field. In recent decades, substantial amount of research has examined Chinese EFL learners' beliefs about language learning (e.g., Zhang and Cui, 2010; Pan and Block, 2011). However, most of these studies are cognition-oriented (Gabillon, 2005).

The cognitive perspective adopts beliefs as a isolated phenomenon and ignores the dynamic and contextualized nature of beliefs which are concerned with socio-cultural contexts (Amuzie and Winke, 2009; Yang and Kim, 2011). Therefore, a socio-cultural approach is much needed for understanding the complex change process of learners‘ beliefs. This book enriches the scholarship of research on learners‘ beliefs from a socio-cultural approach.

As for theoretical contribution, this study has made improvements to the previous analytical socio-cultural framework adapted from Layder (1993, 2006), Gao (2010), and Lamb (2013). Specifically, this extended socio-cultural framework integrates agency, mediation, contextual conditions, and their dialectical relationship in a socio-cultural perspective to illuminate the dynamic nature of beliefs. This adapted analytical framework has revealed that changes in contextual conditions are to a large degree responsible for the shifts in the participants' BLLs. It is also validated that learner agency is of practical value in understanding the development of learner variables. In addition, extracurricular activities, material conditions and cultural artifacts are also important factors that might have influenced the participants' BLLs (LaScotte et al., 2022). Lastly, it has been suggested that the interaction between agency and context plays a significant role in learner‘s BLLs. In other words, learners internalize the influence of contextual realities and accordingly mediate and regulate their BLLs by exercising their agency. We can therefore say that this newly-adapted framework enables us to understand BLLs more properly and comprehensively.

Practically, it is pedagogically implicative for EAP practitioners, educators and administrators to acknowledge that the development of learner BLLs is complex in nature due to the influence of learner agency and context. To better understand the complexity and help EFL learners, it is necessary for EAP practitioners to raise these learners‘ awareness of a particular learning context. Therefore, they can better exert their agency in terms of strategic learning, micro-political, socio-cultural, and intrapersonal capacity. For example, learners are encouraged to be informed of learning objectives, learning conditions and facilities in the orientations. Moreover, dynamic formative assessment method is advocated due to its strong influence upon the participants' beliefs in learning English. In addition, the book has foregrounded the significance and necessity of paying attention to the interplay between agency and contextual realities. Thus, it is suggested that educators and administrators should take into full consideration the learners' needs to survive in an EMI academic context which exerts great influence on their BLLs when designing the EAP curriculum. They could also provide extracurricular activities and continuing language guidance, support, and encouragement for learners in the development of their beliefs to learning EAP in the EMI context.

Methodologically, this study employs a mixed methods paradigm which reasonably overcomes the limitations of purely quantitative or qualitative approaches, for instance, being too general or unrepresentative. Specifically, in this study, the questionnaire provides a general understanding of the learners' beliefs, motivation, and strategy use. Then, it is complemented by the semi-structured interviews, offering further detailed and in-depth insights into these learner variables. Therefore, this study is encouraging and valuable for providing an insightful empirical research paradigm that provides reference to future research in this area. In addition, the author conducted large scale longitudinal research, involving 1,935 students in Questionnaire Survey 1 and 2 and 24 students in interviews at two research stages. The sufficient samples and dynamic observation could add to the generalizability and guarantee the credibility of the results.

As previously stated, this manuscript is worthy of being commented on and recommended to more readers. Admittedly, there is some room for improvement in this book. Firstly, the sole source of the research participants is one EMI university. The results yielded from the sampled university could not represent all the kinds of existing EMI institutions in China. Secondly, as for the research design, interviews alone may be inadequate for the triangulation of the quantitative data. Therefore, it is advised that more qualitative data are collected(e.g., self-reports data) to improve the reliability. In addition, the findings will be more robust and comprehensive if inferential analysis (e.g., exploratory factor analysis) is adopted. Thirdly, this study echoes the call for more research with an inclusion of other elements such as learners' motivation and beliefs in learner agency (Gao, 2010). However, changes occur during the investigation of learners beliefs during multilingual contexts (Pirhonen, 2022), which becomes a new norm in language learning studies. It is recommended that future studies can take these issues into consideration to a wider variety of EMI contexts.

Undoubtedly, the strengths of this book far outweigh its shortcomings. The book is informative, insightful and inspirational with its theoretical, practical and methodological values. With thoughtful reading, readers will hopefully gain a deeper understanding of the disposition of learner beliefs among Chinese EFL learners, and acquire a fuller scenario of the interplay between learner agency and context from a critical socio-cultural perspective. Therefore, this book is strongly recommended to a wider readership including academics, administrators, educators and those who have keen interests in learners' belief from a more kaleidoscopic perspective.

## Author contributions

LQ drafted the paper. ZS helped LQ to select the commented book and provided insights and suggestions during his writing. SZ did the revision for the text. All authors contributed to the article and approved the submitted version.

## Conflict of interest

The authors declare that the research was conducted in the absence of any commercial or financial relationships that could be construed as a potential conflict of interest.

## Publisher's note

All claims expressed in this article are solely those of the authors and do not necessarily represent those of their affiliated organizations, or those of the publisher, the editors and the reviewers. Any product that may be evaluated in this article, or claim that may be made by its manufacturer, is not guaranteed or endorsed by the publisher.
